# Mechanical properties and mechanism of soil treated with nano-aqueous adhesive (NAA)

**DOI:** 10.1038/s41598-022-19108-5

**Published:** 2022-08-29

**Authors:** Wei Huang, Jiaxin Du, Haoqiang Lai, Qingxiu Zhang, Cuiying Zhou, Zhen Liu

**Affiliations:** 1 School of Civil Engineering, Sun Yat-Sen University, No.135 XinGangXiLu, Guangzhou, 510275 China; 2 Guangdong Engineering Research Centre for Major Infrastructure Safety, School of Civil Engineering, Sun Yat-Sen University, Guangzhou, 510275 China

**Keywords:** Environmental sciences, Engineering, Civil engineering

## Abstract

The loose structure and low mechanical strength of the surface soil make it vulnerable to damage under erosion conditions. Slope ecological protection is one of the effective methods to improve the stability of slope soil. Although it has been proved that polymer modified materials can effectively improve the soil properties and the environmental protection effect of slope, so far, the improvement mechanism has not been fully understood, especially the chemical mechanism of the material on the enhancement of soil mechanical properties is not clear. In the present study, the effects of nano-aqueous adhesive (NAA) on unconfined compressive strength, shear strength and aggregate characteristics of soil were studied by a series of laboratory experiments. The results show that NAA can increase the strength, aggregate number and stability of the soil, to effectively improve the stability of surface soil. In addition, through infrared spectroscopy and SEM test, it was found that NAA molecules were mainly distributed in the interlayer position of flaky clay minerals, mainly connected with clay minerals through hydrogen bonds, thereby effectively enhancing the cohesion of soil particles.

## Introduction

There are a large number of clay slopes in southern China. Under the action of rainfall, the surface soil is vulnerable to erosion due to its loose structure and low mechanical strength, resulting in soil and water loss on the slope. In severe cases, it even leads to slope damage, threatening the safety of buildings near the slope^1–3^. Therefore, it is necessary to protect the surface soil to reduce runoff erosion on the slope.

The traditional slope protection technologies mainly include lattice beam, shotcrete and concrete retaining walls^4,5^, there are often shortcomings such as high project cost, poor environmental performance, and destroyed original landscape. With the increasingly stringent environmental requirements, plant-based slope ecological protection technology has attracted more and more attention. Ecological interventions have been proven an effective method to improve the erosion resistance of slopes^6,7^, including artificial turf, soil spraying and planting bags, etc.,^8–10^. However, in the early stage of plant germination and growth, the roots and leaves have not been completely covered on the whole slope, and cannot form effective protection for the slope. The exposed soil is prone to damage under the erosion of runoff or wind^11^, resulting in seeds and seedlings being unable to grow normally, which leads to poor ecological protection of slope^12^. Therefore, it is necessary to improve the slope soil so that it can remain stable at the initial stage of plant growth and provide a stable growth environment for plants^1,13,14^.

At present, the commonly used soil improvement materials can be divided into three categories: biological, inorganic and organic. The biological improvement method is: microbially induced calcium carbonate precipitation (MICP)^15,16^, its essence is to use the urease produced by bacteria to decompose urea, to induce carbonate ions to combine with metal cations to form gel crystals. Because of its long curing process and complex operation, it is not suitable for slope ecological protection. It is generally used to improve sand liquefaction, concrete crack repair and tailings solidification; Inorganic soil improvement materials are mainly cement, gypsum, fly ash, etc.^17–23^, although it can effectively improve the mechanical strength of soil, the improved soil has the problems of high strength, large stiffness, poor permeability and residual toxic substances, which are not conducive to plant growth. It is commonly used to strengthen foundation and embankment; Organic soil improvement materials are further divided into natural and semi-natural polymers (e.g., straw polysaccharides cellulose, lignin and resin gum, etc.)^24−26^ and synthetic polymers (e.g., polyacrylic acid, vinyl acetate maleic acid and polyvinyl alcohol, etc.)^27,28^. They have a certain degree of soil improvement effect, and generally do not affect the environment. Therefore, it has received extensive attention.

To date, different types of polymer soil improvers have been developed^29,30^. For example, the polymer modifiers materials developed by Kim et al.^31^ have good soil water retention and show good improvement effects on soil physical and chemical properties. Huang and Liu^32^ studied the application effect of polymer modifiers in improving soil structure, improving slope erosion resistance and restoring slope ecology through laboratory tests. In addition, polymer modifiers also show good application prospects in improving fertilizer utilization rate and saving water resources^33,34^. The effect of polymer modifiers on the mechanical properties of soil is also focused. Azzam^35^ studied the effect of polymer modifiers on the mechanical properties of soil. The results showed that the tensile and shear strength of soil samples increased with the increase of polymer modifier content. In addition, the polymer modifier also improves the mechanical properties of soil by improving the particle composition, microstructure and pore size distribution of the soil^36,37^.

While significant progress has been made in understanding the improved physical, mechanical and ecological properties of the polymer modifier, the research on the mechanism of improvement is limited. Further, the mechanical properties of unsaturated soils are affected by many factors and the problem could become further complicated under unilateral free conditions^38^. Although some experimental efforts have been made^39,40^, the change of soil mechanical properties after the application of polymer modifiers has not been fully understood, especially the chemical mechanism of the material on the enhancement of soil mechanical properties is not clear, and therefore further experimental studies are required. In this paper, we use nano-aqueous adhesive (NAA) as the research object, which is a kind of hydrolysis resistant modified polyester. It has the characteristics of large molecular weight, high viscosity, small particle size and good emulsion stability. After adding it to the soil, it does not affect the growth of vegetation, and the degradation products are CO_2_ and H_2_O, which is ecological soil improvement material^41,42^. In the present study, a series of experimental studies were carried out to investigate the mechanical properties and mechanism of soil treated with nano-aqueous adhesive (NAA) and its improvement mechanism were conducted to help people better understand the mechanism and improvement effect of the NAA from microscopic scale, which can provide ideas and theoretical support for the study of slope soil improvement.

## Materials and method

### Materials

**Figure 1:**
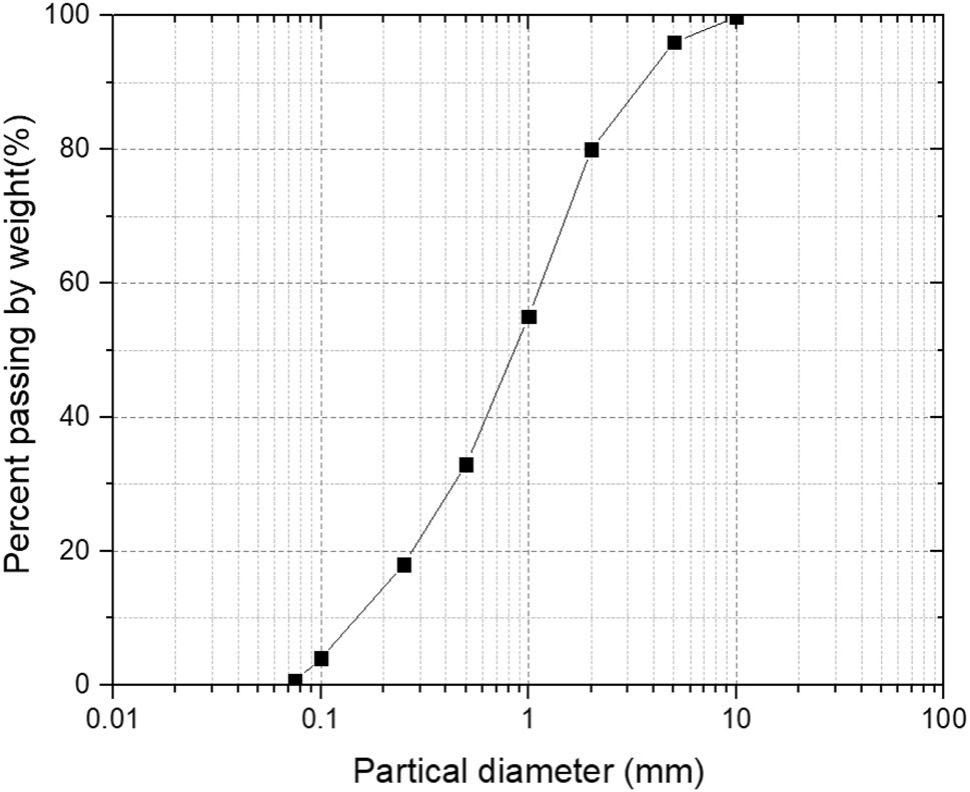
Grading curve.

The test soil was taken from Guangzhou City, Guangdong Province, and the soil depth was 0–30 cm. It was a common silty clay in South China. The soil bulk density 1.42 g/cm^3^, particle density 2.64 g/cm^3^, liquid limit 28.75%, plastic limit 16.32%, maximum dry density 1.81 g/cm^3^, optimum moisture content 15.8%^13^. The grading curves of the soil is shown in Fig. 1.

NAA (polyvinyl acetate, [CH_2_CHCOOCH_3_]_n_) is a white milky colloid with a pH value of 6–7, a density of 1.01 g/cm^3^, and viscosity of 8000–10,000 mPa·S. In this paper, NAA is used to reinforce the soil to improve the strength and stability of the soil.

Polymer (polyvinyl acetate) is generated by the polymerization of vinyl acetate. The preparation process of NAA was as follows^43^: Monomer material was added to the polymerization kettle with agitator and reflux condenser and preheated. Then initiator was added according to the ratio. The polymerization was carried out for several hours at a certain temperature. After the polymerization, rectification was carried out to remove impurities. NAA produced by this method is cheap, and the price is far lower than the existing products.

**Figure 2:**
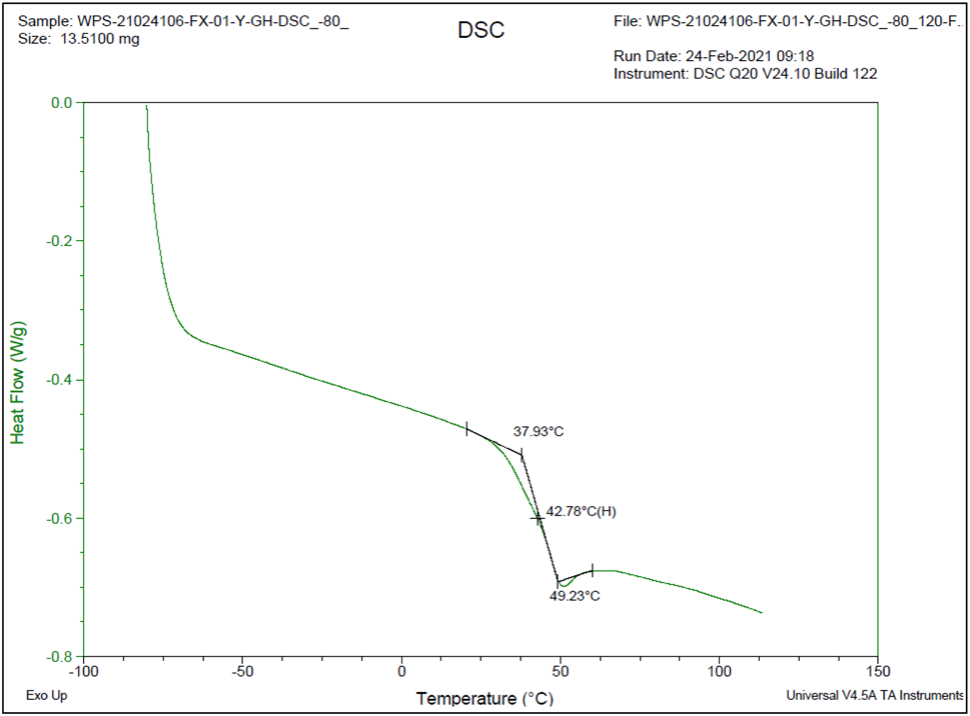
Differential scanning calorimetry (DSC) analysis results.

The physical and chemical properties of NAA were studied by differential scanning calorimetry (DSC), and the heat absorption and release during phase transition, glass transition and chemical reaction were measured (Fig. 2).

DSC analysis showed that the glass transition temperature of the NAA was 42.8 °C. The NAA is mainly used to improve the ability of soil particles to resist erosion by rainfall. Under the current climatic conditions of the earth, it is difficult for the soil to reach that temperature during rainfall, indicating that the NAA can maintain the stability of the soil well under natural working conditions.

### Materials ratio

According to the previous research and engineering practice experience of our team, the dosage of NAA was designed to be 0%, 0.25%, 0.5%, 0.75% and 1% (i.e., NAA mass/water mass × 100%), respectively. The test group with 0% dosage was the control group (CK). The curing time was designed to 0, 1, 2, 3, 5, 7 days in turn. However, through the unconfined compressive strength test, it was found that the change of soil strength on the first to third days was basically linear, so the test on the second day was cancelled in the shear test. As shown in Table 1, the dosage of NAA in each test and the curing time of the sample are listed.

**Table 1:** The dosage of NAA and curing time.

Test types	The dosage of NAA(%)	The curing time(day)
Unconfined compressive strength	0,0.25, 0.5,0.75,1	0, 1, 2, 3, 5, 7
Shear strength		0, 1, 3, 5, 7
Aggregate characteristic		7
Infrared spectroscopic	1	7
Scanning electron microscope (SEM)		

### Test method

After drying and crushing, the soil was screened and the soil with a size less than 2 mm was left for the test. Mix NAA with water and spray evenly on soil (water content is 17%). The control group was sprayed with the same amount of water. The soil was filled into an aluminium box with a length of 1000 mm, a width of 500 mm and a depth of 100 mm, and use the static pressure method to make the dry density of the soil reach the design value (1.7 g/cm^3^), which was consistent with the original soil in the study area. The sample density was measured by the cutting ring method (GB/T 50,123–1999, i.e. a national criterion for geotechnical tests in China which was set based on ASTM standards) to ensure the uniformity and representativeness of the sample. Then put it in a constant temperature curing instrument, and the temperature was set to the average temperature and humidity of spring in southern China (temperature is 25 °C, humidity is 60%).

**Figure 3:**
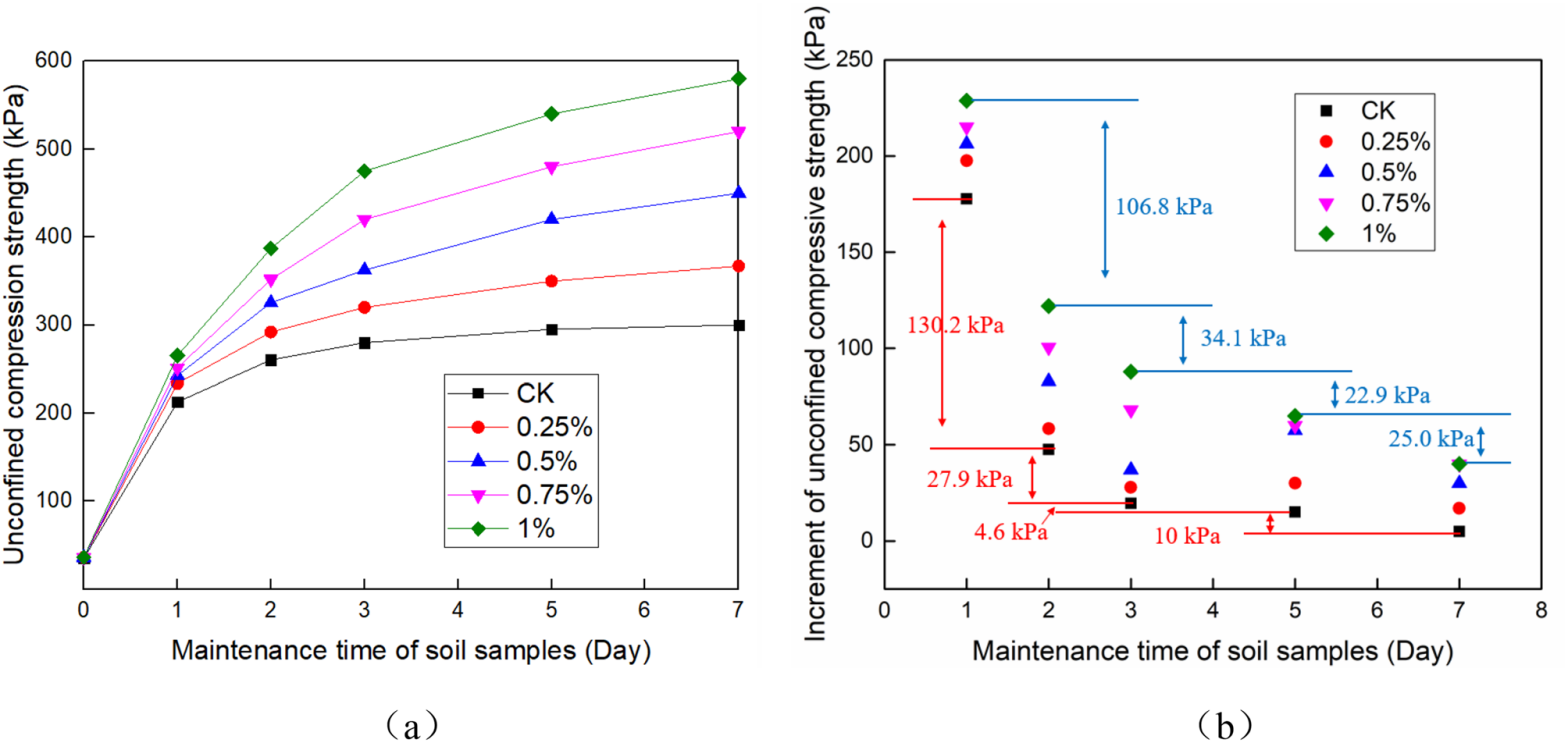
Unconfined compressive strength of the soil under different curing ages. (**a**)The strength of the soil, and (**b**) The strength increment of the soil.

**Figure 4:**
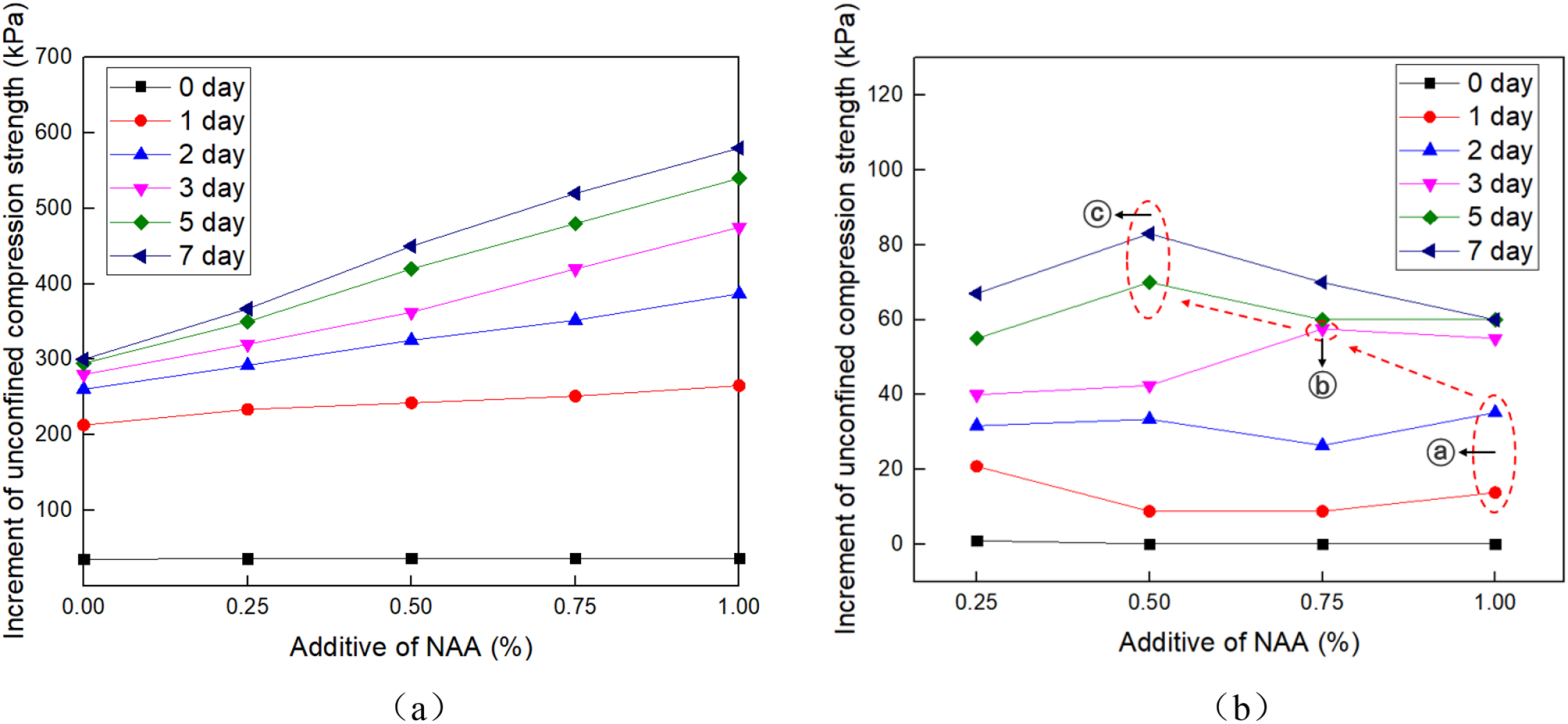
Unconfined compressive strength of the soil under different NAA content. (**a**)The strength of the soil, and (**b**) The strength increment of the soil.

Unconfined compressive strength test was carried out according to test methods of soil (GB/T 50,123–1999, i.e. a national criterion for geotechnical tests in China which was set based on ASTM standards). An appropriate amount of undisturbed soil was taken and cut into standard samples with an inner diameter of 39.1 mm and a height of 80 mm. The YYW-2 strain-controlled unconfined pressure apparatus produced by Nanjing Soil Instrument Factory was used for the test, and the strain rate was 2.0 mm/min. Each group of tests was conducted three times, and the average value was taken as the test result. The results are shown in Figs. 3 and 4.

**Figure 5:**
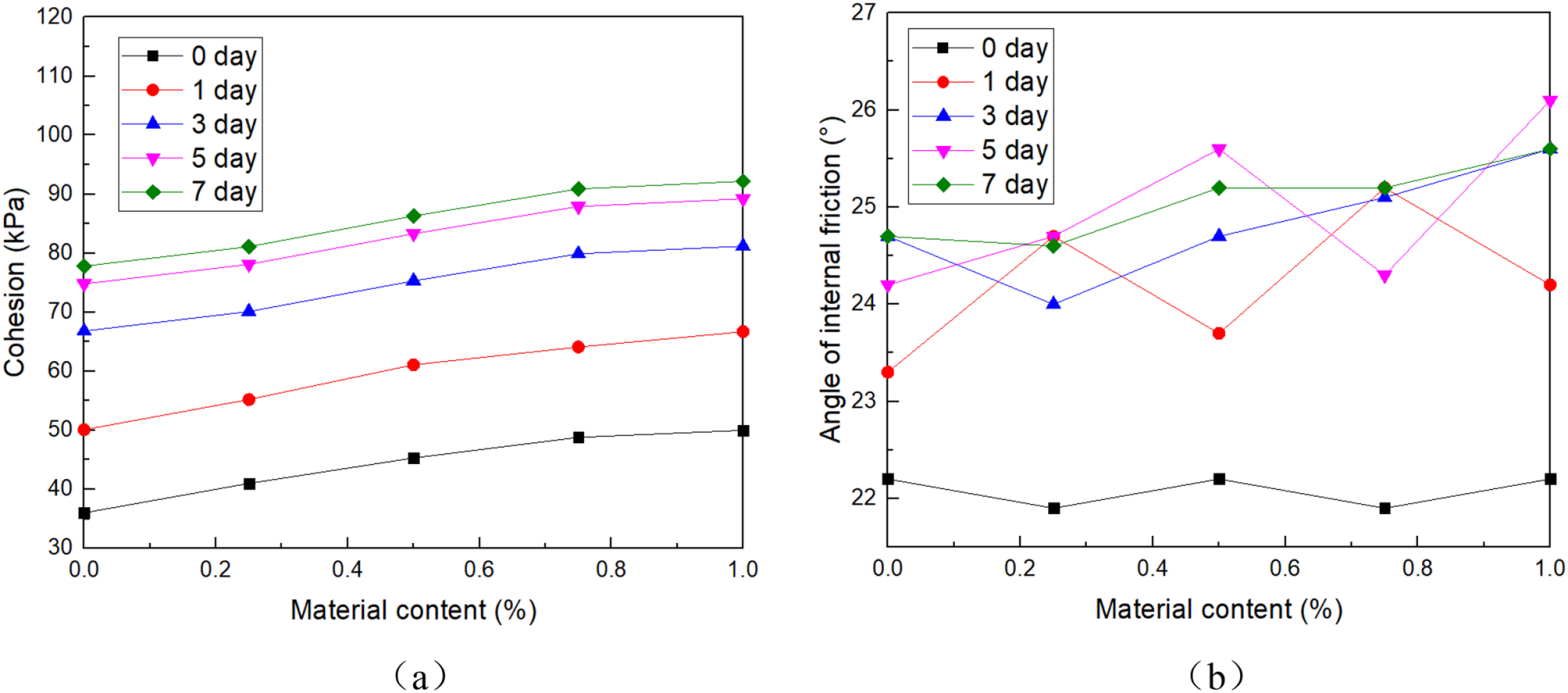
Cohesion and internal friction angle of the soil under different NAA content. (**a**) Cohesion, and (**b**) Angle of internal friction.

**Figure 6:**
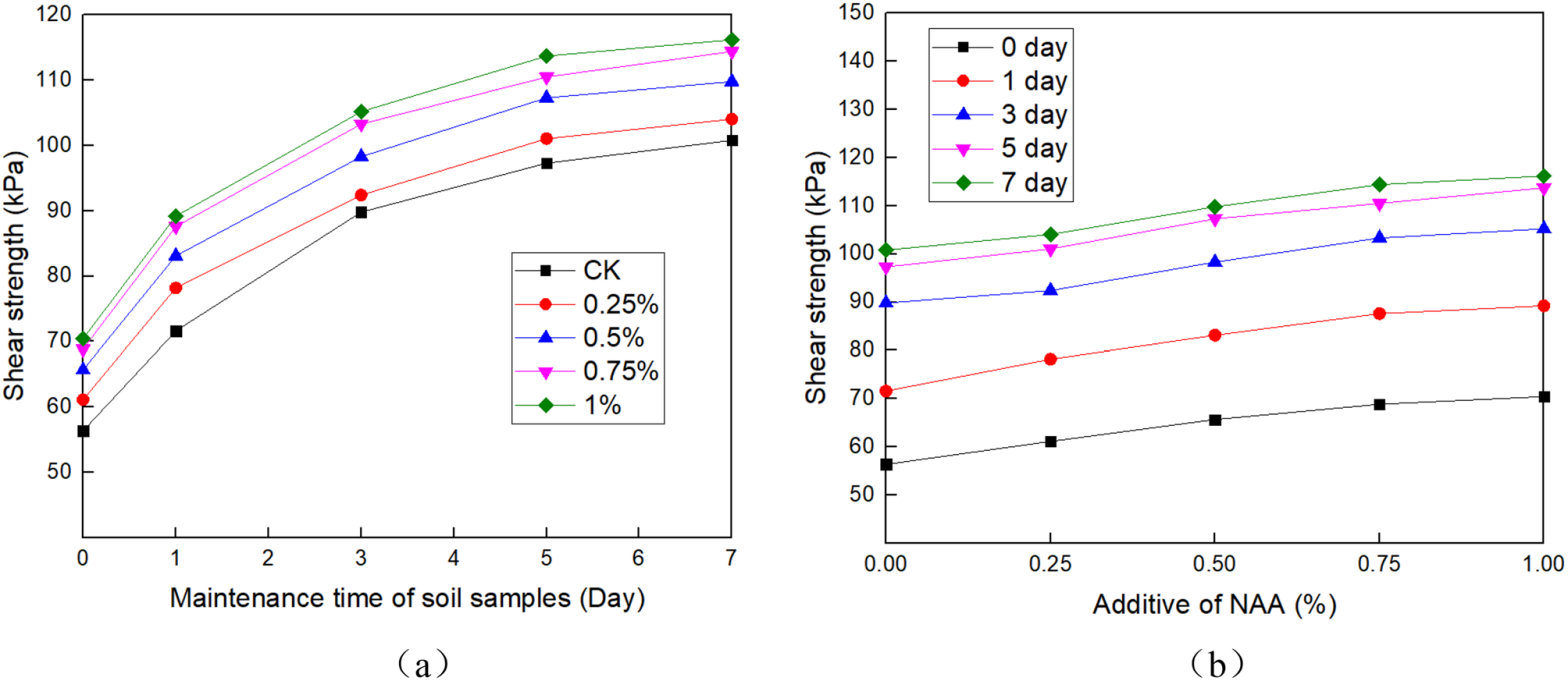
Shear strength under 50 kPa axial compression. (**a**) Under different curing ages, and (**b**) Under different NAA content.

The shear test was carried out according to test methods of soil (GB/T 50,123–1999, i.e. a national criterion for geotechnical tests in China which was set based on ASTM standards). According to actual engineering conditions, the shear strength of the samples was measured by an unconsolidated and undrained shear test (UU test). The test instrument is ZJ four-way strain-controlled direct shear apparatus produced by Nanjing Soil Instrument Factory. The vertical loads are 50, 100, 200 and 400 kPa respectively, and the strain rate is 0.8 mm/min. Each group of tests was conducted three times, and the average value was taken as the test result. The results are shown in Table 2, Figs. 5 and 6.

**Table 2:** Cohesion and internal friction angle.

NAA (%)	Parameter type	Curing time(day)				
		0	1	3	5	7
0	Cohesion	36.0	50.1	66.8	74.8	77.8
	Angle of internal friction	22.2	23.3	24.7	24.2	24.7
0.25	Cohesion	41.0	55.2	70.1	78.1	81.1
	Angle of internal friction	21.9	24.7	24.0	24.7	24.6
0.5	Cohesion	45.3	61.1	75.3	83.3	86.3
	Angle of internal friction	22.2	23.7	24.7	25.6	25.2
0.75	Cohesion	48.8	64.1	79.9	87.9	90.9
	Angle of internal friction	21.9	25.2	25.1	24.3	25.2
1	Cohesion	50.0	66.7	81.2	89.2	92.2
	Angle of internal friction	22.2	24.2	25.6	26.1	25.6

Soil aggregates can be divided into water-stable aggregates and non-water-stable aggregates. Among them, water-stable aggregates refer to the soil structure that does not immediately disperse after water immersion, which can maintain the soil structure morphology to a certain extent; Non-water-stable aggregates cannot maintain soil structure after water immersion. Mechanical screening methods can be used to determine different types of soil aggregates. The content of non-water stable aggregates was determined by the dry sieve method, and the content of water-stable aggregates was determined by the wet sieve method (Yang and Lu^44^; Li et al.^45^). According to the method recommended by Ning et al.^46^ and Shi and Shao^47^ the aggregate stability index (ASI) can be obtained by analyzing the test results of the dry and wet sieve.

Among them, the pore size of the sieves was: 5, 2, 0.5, 0.25, > 0.25 mm. When wet sieving, the set of sieves was shaken up and down in the water for 30 min at the frequency of 30 times min^–1^. The contents of non-water-stable aggregate (${M}_{1}$ (g/kg)) and water-stable aggregates (${M}_{2}$ (g/kg)) were calculated according to the following formula:

1 $${M}_{1}=\frac{{m}_{1}^{,}}{{m}_{1}}\times 1000$$

${m}_{1}^{,}$–Quality of non-water-stable aggregate at all levels, g; ${m}_{1}$–Quality of air-dried soil samples, g.

2 $${M}_{2}=\frac{{m}_{2}^{,}}{{m}_{2}}\times 1000$$

${m}_{2}^{,}$–Quality of water-stable aggregate at all levels, g;${m}_{2}$–Quality of air-dried soil samples, g.

Combined dry and wet sieve methods to simulate the process of rain erosion and calculate the aggregates stability index (*ASI*)^46,47^:

3 $$ASI={X}_{1}+{X}_{2}+{X}_{3}+{X}_{4}+{X}_{5}$$

where, ${X}_{1}$, ${X}_{2}$, ${X}_{3}$, ${X}_{4}$, and ${X}_{5}$ represent the probability of soil aggregates stored at > 5, 2, 0.5, 0.25, > 0.25 mm sieve levels (stability coefficient), respectively.

The mean weight diameter (MWD) of the soil is calculated using the following equation:

$$MWD=\sum_{i=1}^{n}{x}_{i}{w}_{i}$$

where ${x}_{i}$ is the average diameter of the sieved agglomerates in any size range, and ${w}_{i}$ is the weight of the agglomerates in any size range as a fraction of the dry weight of the soil sample. Three parallel controls were set up for each group, and the results were averaged to calculate the standard error of the data.

**Figure 7:**
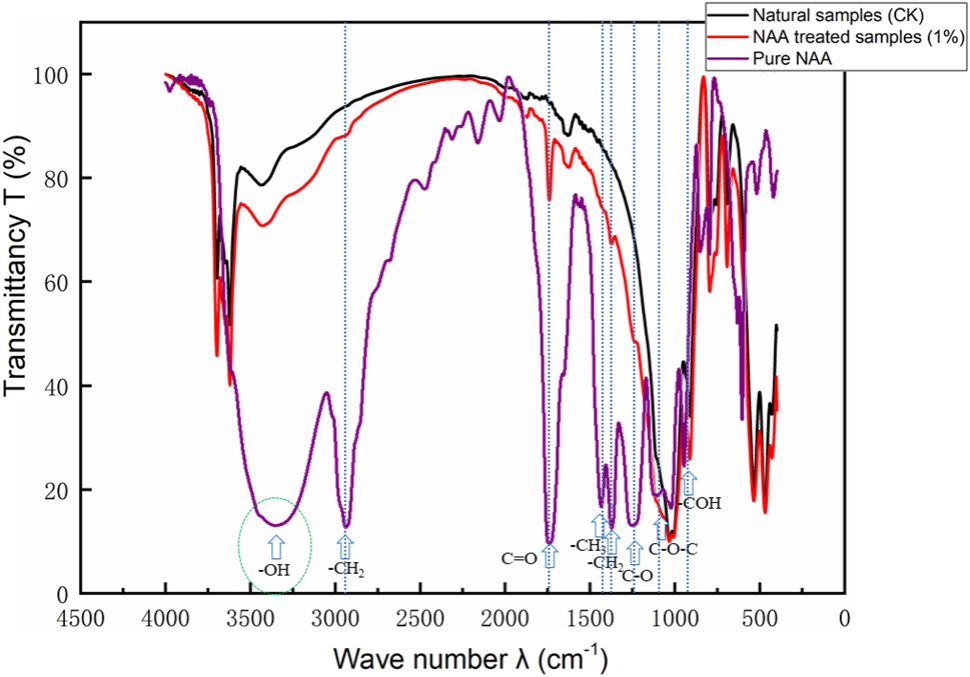
Results of infrared spectroscopy.

A small amount of natural soil and improved soil was taken and placed in an oven for 24 h (Temperature 105 °C) to make it completely dry to avoid the influence of free water. Then the Fourier transform infrared spectroscopy-microscope instrument (Model: Nicolet6700 – Contiuμm, wavenumber range: 400–7500 cm^−1^, resolution: 0.09 cm^−1^) of the test centre of Sun Yat-sen University was used to test, and the test curve was processed by OPUS software to reduce the influence of human factors on the spectrum. The results are shown in Fig. 7.

**Figure 8:**
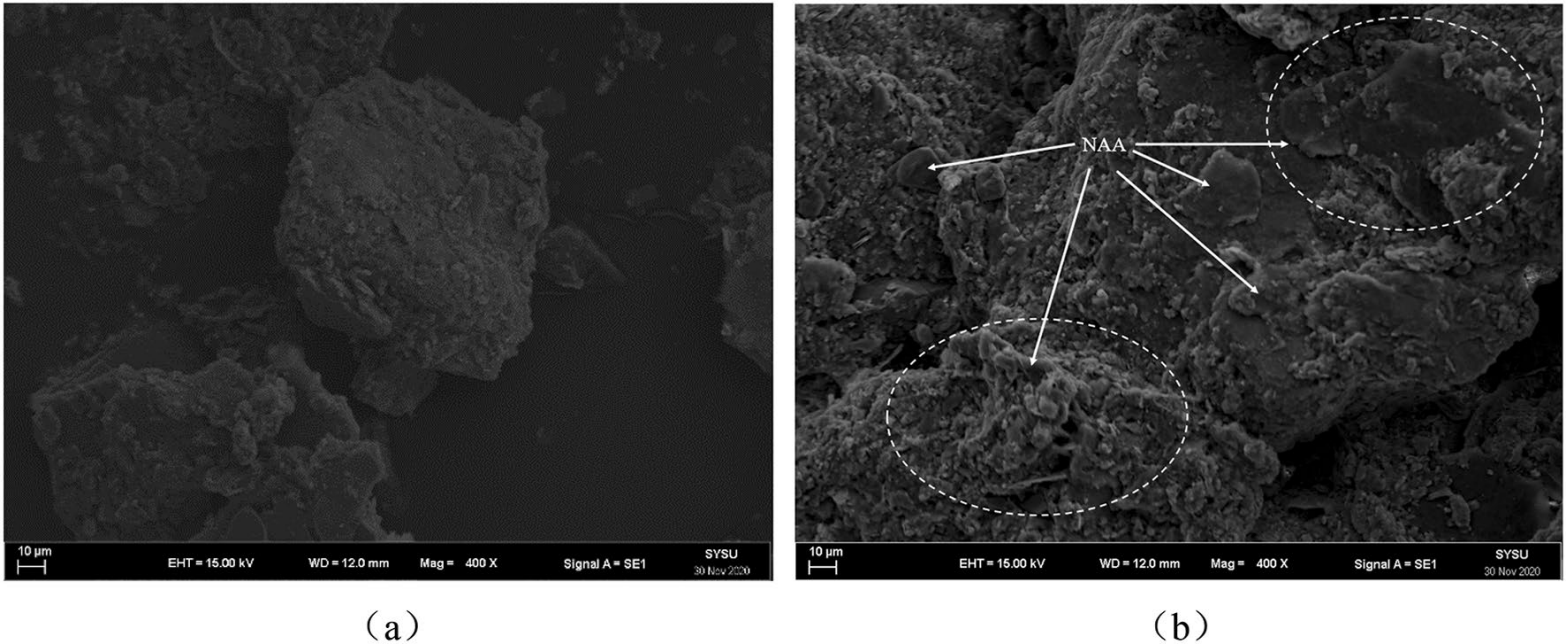
SEM of the microstructure of soil samples. (**a**) Natural soil ($\times$ 400), and (**b**) NAA treated soil.

A small amount of natural soil and improved soil were fixed on the sample table after freeze-drying. Gold was sprayed on the surface of the soil to improve the quality and resolution of the image. Then the thermal field emission scanning electron microscope (Model: Gemini 500, resolution: 0.6 nm) was used in the Testing Center of Sun Yat-sen University. The results are shown in Fig. 8.

## Results

### Unconfined compressive strength

Figure 3 shows the unconfined compressive strength of the soil under different curing ages. The results show that with the increase of curing time, the unconfined compressive strength of each group of samples increases (Fig. 3a). When the curing time was 0 days, the unconfined compressive strength of each group of samples was very close, between 35.1 and 36.4 kPa. With time, the difference of each group gradually appears and continues to increase. The higher the NAA content is, the greater the strength is. After 7 days of curing, the unconfined compressive strength of each group with NAA content from 0 to 1% was 300.2 kPa, 367 kPa, 452 kPa, 520 kPa and 580 kPa, respectively.

It can be seen from Fig. 3b that the higher the NAA content is, the greater the strength increment is. With the passage of time, the strength increment of each group of samples is gradually reduced. Among them, the strength increase of each group was the largest on the 1 day, reaching 177.7 kPa–228.8 kPa. In the next 2–7 days, the strength increases of each group were 47.5–122.0 kPa, 19.6–87.9 kPa, 15–65 kPa and 5.1–40 kPa, respectively. The results show that the improvement effect of NAA on soil strength is more obvious in the early stage.

Figure 4 shows the unconfined compressive strength of the soil under different NAA content. The results show that the material content has little effect on the unconfined compressive strength of the sample when the curing time is 0 days. When curing is 1–7 days, the unconfined compressive strength of the sample gradually increases with the increase of the material content (Fig. 4a).

The change of NAA content will affect the strength increment of soil. It can be seen from Fig. 4 (b) that with the increase of NAA content, the time of strength increment increase will advance. Circle (a) corresponds to the NAA content of 1%, the maintenance time is 1–2 days. Circle (b) corresponding to the NAA content of 0.75%, the maintenance time is 3 days. Circle (c) corresponding to NAA content of 0.5%, maintenance time of 5–7 days. This phenomenon reflects the positive effect of NAA on the improvement of soil strength. The more NAA content is, the faster the strength is improved.

### Shear strength

Figure 5 and Table 2 shows the shear strength indexes (cohesion and internal friction angle) of the soil under different NAA content. The results show that the cohesion increases with the increase of curing time. When curing for 0 days, the cohesion of each group with NAA content from 0 to 1% was 36.0 kPa, 41.0 kPa, 45.3 kPa, 48.8 kPa and 50.0 kPa respectively. When curing is 7 days, it had increased to 77.8 kPa, 81.1 kPa, 86.3 kPa, 90.9 kPa and 92.2 kPa, with an increase of 116.2%, 97.9%, 90.5%, 86.3% and 84.4%, respectively. Similarly, the cohesive increases with the increase of NAA content: Taking the 3 days as an example, compared with natural soil with 0% content, the cohesive of samples with 0.25%, 0.5%, 0.75% and 1% content increased by 4.94%, 12.72%, 19.61% and 21.56%, respectively. In other curing times, there are the same rules.

From the internal friction angle of Fig. 5 and Table 2, it can be seen that the internal friction angle of the sample almost does not change with the increase of NAA content. With the extension of curing time, the internal friction angle increases only slightly.

NAA is mainly used to improve the surface soil of the slope, and the overlying pressure is small. To better meet the engineering site conditions, the stress–strain curve under 50 kPa axial compression is selected for analysis. Figure 6 shows the shear strength of soil at different curing ages under different NAA content. The results show that the shear strength increases with the increase of curing time. When curing for 0 days, the shear strength of each group with NAA content from 0 to 1% was 56.7 kPa, 61.8 kPa, 65.9 kPa, 68.6 kPa and 70.5 kPa respectively. When curing is 7 days, it had increased to 101.6 kPa, 104.8 kPa, 109.8 kPa, 114.7 kPa and 114.9 kPa, with an increase of 79.19%, 69.58%, 66.61%, 67.20% and 62.98%, respectively.

Similarly, the shear strength increases with the increase of NAA content: Taking the 3 days as an example, compared with natural soil with 0% content, the shear strength of samples with 0.25%, 0.5%, 0.75% and 1% content increased by 2.90%, 9.47%, 15.03% and 17.15%, respectively. In other curing times, there are the same trends.

### Aggregate characteristic

Table 3 shows the content of aggregates at all levels. The results show that under the condition of dry sieving, NAA only affected the content of improved soil in groups with particle size ≥ 5 mm, which was higher than that of natural soil, and the trend of other components was not obvious. Under the condition of wet sieving, NAA significantly increased the content of soil aggregates. The proportion of soil aggregates (particle size ≥ 0.25 mm) of each group with NAA content from 0 to 1% was 59.9%, 83.9%, 93.3%, 98.8% and 98%, respectively. Previous studies^48^ have shown that increasing the proportion of soil aggregates can improve soil structure, improve soil stability and promote plant growth.

**Table 3:** Particle size distribution of soil aggregates.

NAA(%)	Sieving method	Pore size of the sieves					MWD (%)	Aggregates stability index (ASI)
		≥ 5 mm	2–5 mm	0.5–2 mm	0.25–0.5 mm	< 0.25 mm		
0	Dry	86.34 ± 0.2	6.45 ± 0.1	1.53 ± 0.1	4.21 ± 0.2	1.38 ± 0.1	5.88	0.1057
	Wet	0.20 ± 0.0	1.90 ± 0.1	22.80 ± 0.2	35.00 ± 0.1	40.10 ± 0.0	0.56	
0.25	Dry	87.53 ± 0.1	7.99 ± 0.1	1.08 ± 0.2	2.39 ± 0.1	0.99 ± 0.1	5.99	0.1059
	Wet	1.90 ± 0.0	5.30 ± 0.1	62.30 ± 0.0	14.40 ± 0.1	16.10 ± 0.2	1.17	
0.5	Dry	88.10 ± 0.1	9.58 ± 0.0	0.77 ± 0.1	0.20 ± 0.1	1.35 ± 0.0	6.07	0.1125
	Wet	68.50 ± 0.2	17.40 ± 0.1	5.60 ± 0.0	1.79 ± 0.1	6.71 ± 0.1	5.15	
0.75	Dry	86.88 ± 0.2	8.75 ± 0.0	1.49 ± 0.1	2.27 ± 0.0	0.68 ± 0.1	5.98	0.1141
	Wet	66.20 ± 0.1	15.20 ± 0.1	13.40 ± 0.0	4.00 ± 0.1	1.20 ± 0.1	5.02	
1	Dry	90.86 ± 0.1	5.68 ± 0.1	1.45 ± 0.0	0.58 ± 0.2	1.43 ± 0.0	6.13	0.1088
	Wet	30.00 ± 0.1	25.80 ± 0.1	33.20 ± 0.0	9.00 ± 0.1	2.00 ± 0.1	3.30	

Average value ± standard error.

It can be seen from Table 3 that compared with the dry sieving, the content of soil aggregates in each group in the wet sieving was significantly reduced, indicating that hydraulic erosion destroyed the soil structure, resulting in softening failure of large aggregates with the low internal bond strength and disintegration into smaller aggregates^44^. Compared with natural soil (0%), NAA can effectively improve MWD, but the improvement trend is not obvious.

By analyzing the stability coefficients of each group, it can be seen that compared with natural soil (0%), the stability coefficients of each group with NAA content from 0.25 to 1% was increased by 0.19%, 6.43%, 7.95% and 2.93%, respectively. It shows that NAA can effectively improve the water stability of soil aggregates.

### Infrared spectroscopic

Functional groups of samples can be determined by Fourier transform infrared spectroscopy^49−51^. In the pure NAA curve (Fig. 7), the peak at 3380 cm^−1^ is the stretching vibration absorption peak after hydrogen bonding between hydroxyl groups (–OH). 2900 cm^−1^ is the asymmetric stretching vibration peak of C–H in methylene –CH_2_-. The characteristic absorption peak of C = O stretching vibration appeared at 1730 cm^-1^. The stretching vibration absorption peak of C–H in -CH_3_ appeared at 1430 cm^−1^. 1375 cm^−1^ is the bending vibration absorption peak of C–H in –CH_2_. 1239 cm^-1^ is the stretching vibration absorption peak of the C–O bond in the ester group. Strong absorption near 1100 cm^−1^ is the stretching vibration peak of C–O–C (ester). Strong absorption near about 930 cm^−1^, reflecting –COH (carboxylic acid) out-of-plane bending.

### Electron microscope scan

Figure 8 shows the scanning electron microscope (SEM) of soil. It can be seen that in the natural soil samples (Fig. 8a), the soil particles are loose and broken, the edges and corners are clear, the pore spacing between particles is large, and the particle surface is relatively smooth. After adding NAA into the soil (Fig. 8b), the loose particles became less and the surface was rough. Careful observation can be found that the NAA mainly exists in the layer of flake soil particles, which is the same as the infrared spectrum test results. On the one hand, the NAA between layers increases the cohesive force between particles and promotes the aggregation of particles. On the other hand, fine soil particles adhere to the surface of large aggregates and increase the roughness of aggregates.

## Discussion

### Effect of NAA on mechanical properties of soil

The unconfined compressive strength and shear strength tests show that NAA can effectively improve the mechanical properties of soil, but with the different curing times and dosage^39^, the improvement effect is different. It can be seen from Fig. 3 that the strength of soil increases with the extension of curing time, and the strength increment gradually decreases with time. It indicates that curing is very important for improving the mechanical properties of soil. In practical engineering, continuous construction should be avoided. After NAA is applied, it is necessary to cure for a period of time.

It can be seen from Figs. 3 and 4 that the higher the NAA content is, the greater the strength improvement rate of the sample is, and the longer the time to reach the peak strength is, the greater the peak strength is. Therefore, when NAA is used in slope ecological protection engineering, it is necessary to comprehensively consider the time and economic benefit. When the construction period is tight, a reasonable increase of NAA content helps to accelerate the improvement of soil strength and achieve the design strength faster.

The shear strength of soil is determined by cohesion and internal friction angle^52^. It can be seen from Figs. 5, 6 and Table 2 that the shear strength, cohesion and internal friction angle increase with the increase of curing time. With the increase of NAA content, only the shear strength and cohesion increased, and the internal friction angle changed slightly^13^. It can be seen that NAA improves soil strength mainly by increasing the cohesion between soil particles.

### Effect of NAA on soil aggregates

The stability of soil aggregates is one of the important factors that affect the stability of soil structure and anti-erosion ability^53^. The analysis showed that NAA improved soil structure mainly by increasing aggregate content and stability. Compared with natural soil, the proportion, stability and mean weight diameter (MWD) of soil aggregates (Particle size ≥ 0.25 mm) treated by NAA were significantly improved. By comparing the aggregate content of dry and wet sieves, it can be seen that under hydraulic erosion, some of the aggregates were softened and disintegrated (NAA-treated and untreated), resulting in a lower soil aggregate content under wet sieves than under dry sieves, but the aggregate content of NAA-treated soils was still significantly higher than that of natural soils, indicating that NAA has a certain degree of water resistance and rainfall erosion does not cause it to fail.

### Effect of NAA on soil microstructure

**Figure 9:**
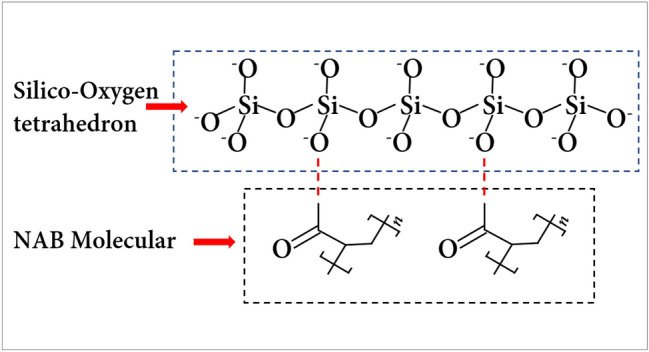
Mechanism diagram of NAA improving clay.

By comparing the infrared spectrum curves of natural soil (CK) and improved soil, it can be seen that except for the hydroxyl (–OH) at 3380 cm^−1^, the other characteristic groups in NAA can be found in the improved soil curve. It shows that hydroxyl is the key group to improve soil properties, indicating that NAA strips the hydroxyl group (–OH) from the molecular chain with the participation of water and combines with (–H) on the clay surface to form hydrogen bonds during the soil modification process^48^. According to the double layer theory^54^, the surface of clay particles is negatively charged. In the process of soil improvement by NAA, with the participation of water, the hydroxyl (− OH) on the molecular chain was removed, making the NAA molecule positively charged^55^. Positive and negative charges attract each other, forming hydrogen bonds at the junction (Fig. 9).

## Mechanism analysis

After NAA was mixed with water, the internal long polymer chain gradually expanded and dispersed into small latex particles in water. The carboxyl group (− COOH) on the molecular chain removed the hydroxyl group (− OH) and the NAA was positively charged. After contact with the negatively charged clay surface, they adsorbed each other and formed a new chemical bond. Through these chemical bonds, the long-chain macromolecules of the polymer wrap the surface of the soil particles and connect with each other to form soil aggregates, and the interconnected long-chain macromolecules form a mesh membrane on the surface of the soil aggregates, which further improved the stability of soil^14,42^. Although the number of polymer modified materials in the soil is small, it can effectively improve soil structure by certain physical and chemical methods, to improve soil stability and corrosion resistance^24,56^. This is also confirmed by the results of scanning electron microscopy (Fig. 8). It can be seen that NAA exists between the soil particles, and the polymer film formed is mainly in contact with the flake clay particles.

**Figure 10:**
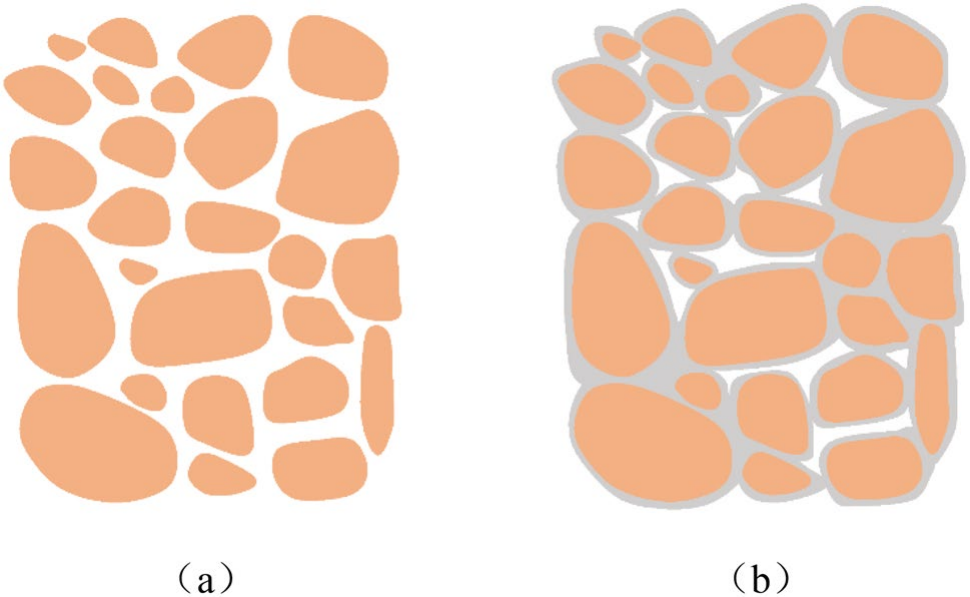
Structure diagram of NAA modified in soil. (**a**) Natural soil, and (**b**) NAA treated soil.

The internal structure of silty clay particles before and after spraying NAA is shown in Fig. 10. The mesh membrane structure formed by NAA will have a porous curing layer on the soil surface, which can effectively improve the compactness, impermeability and mechanical properties of the soil on the one hand. On the other hand, the solidified layer has good wind erosion resistance, water erosion resistance and biodegradability, which can effectively guarantee the growth of plants.

## Conclusion

Based on laboratory tests, the effect of NAA on the mechanical properties of silty clay was studied. The results show:

1. NAA can effectively improve the unconfined compressive and shear strength of soil, but the increase is related to the curing time and material content.
2. NAA can significantly improve the number and stability coefficient of soil aggregates (particle size ≥ 0.25 mm), and enhance the water stability of the soil.
3. NAA and soil particles are mainly linked by hydrogen bonds, so strong adhesion and stability can be obtained.
4. The natural soil without NAA treatment is loose and has a large number of fine particles; After NAA treatment, the number and volume of soil aggregates increased significantly.
5. NAA can effectively improve the soil structure, improve the stability and mechanical properties of soil, and help to improve the erosion resistance of slope surfaces. It is an ideal slope ecological protection material, which can be popularized and applied.

## Data Availability

All data generated or analysed during this study are included in this published article.

## References

[1] Jiang F (2014). Effects of rainfall intensity and slope gradient on steep colluvial deposit erosion in Southeast China. Soil Sci. Soc. Am. J..

[2] Xiang B, Liu E, Yang L (2022). Influences of freezing–thawing actions on mechanical properties of soils and stress and deformation of soil slope in cold regions. Sci. Rep..

[3] Yin, Y., Wang, L., Zhang, W., Zhang, Z. & Dai, Z. Research on the collapse process of a thick-layer dangerous rock on the reservoir bank. *Bull. Eng. Geol. Environ.* **81**, 1–11 (2022).

[4] Ahmadi-Nedushan, B. & Varaee, H. Optimal Design of Reinforced Concrete Retaining Walls using a Swarm Intelligence Technique. *Proc. First Int. Conf. Soft Comput. Technol. Civil, Struct. Environ. Eng.* **92**, 1–12 (2009).

[5] Tiwari N (2022). An experimental study on strength improvement of expansive subgrades by polypropylene fibers and geogrid reinforcement. Sci. Rep..

[6] Sun X, Miao L, Chen R, Wang H, Xia J (2022). Surface rainfall erosion resistance and freeze-thaw durability of bio-cemented and polymer-modified loess slopes. J. Environ. Manag..

[7] Wang G, Han J, Liu D (2003). The carbon isotope composition of C3 herbaceous plants in loess area of northern China. Sci. China Ser. D Earth Sci..

[8] Shaowei W, Degou C, Song L, Jianping Y (2021). Performance test and stability analysis of jute ecological bag on subgrade slope. E3S Web Conf..

[9] Wei C, Huang K, Zhang N, Qin X, Siddique A (2021). Discussion on ecological protection technology of high and steep slope of expressway. IOP Conf. Ser. Earth Environ. Sci..

[10] Bao X, Li H (2020). Study on the Evaluation Method of Subgrade Slope Green Protection Effect in Dry-Hot Valley of Sichuan-Tibet Railway. Math. Probl. Eng..

[11] Ramos-Scharrón CE, Alicea-Díaz EE, Figueroa-Sánchez YA, Viqueira-Ríos R (2022). Road cutslope erosion and control treatments in an actively-cultivated tropical montane setting. CATENA.

[12] Maiti, S. K. Ecological restoration of waste dump generated from an integrated steel plant: A case study. In *Innovative Exploration Methods for Minerals, Oil, Gas, and Groundwater for Sustainable Development* 1st edn, (eds Moitra, A. K. *et al.*) 157–171 (Elsevier Ltd., 2022). 10.1016/b978-0-12-823998-8.00089-2.

[13] Zhou C, Huang W, Qiu S, Liu Z (2021). A quantitative study on the amount of water-retaining agent based on adhesive-modified red bed weathered soil. Bull. Eng. Geol. Environ..

[14] Huang W (2021). Improving soil-water characteristics and pore structure of silty soil using nano-aqueous polymer stabilisers. KSCE J. Civ. Eng..

[15] Tiwari N, Satyam N, Sharma M (2021). Micro-mechanical performance evaluation of expansive soil biotreated with indigenous bacteria using MICP method. Sci. Rep..

[16] Tang CS (2020). Factors affecting the performance of microbial-induced carbonate precipitation (MICP) treated soil: A review. Environ. Earth Sci..

[17] Wang, X. *et al.* Effect of different fly ash additions on the properties of unsaturated soil in open-pit mine dumps. *Arab. J. Geosci.* **14**, 1–10 (2021).

[18] Onyelowe KC, Okafor FO (2015). Review of the synthesis of nano-sized ash from local waste for use as admixture or filler in engineering soil stabilization and concrete production. J. Environ. Nanotechnol..

[19] Zhang J, Fujiwara T (2007). Concrete sludge powder for soil stabilization. Transp. Res. Rec..

[20] Yang B, Zhang Y, Cetin B, Ceylan H (2019). Concrete grinding residue: Management practices and reuse for soil stabilization. Transp. Res. Rec..

[21] Mypati VNK, Saride S, Ph D, Asce M (2022). Feasibility of alkali-activated low-calcium fly ash as a binder for deep soil mixing. J. Mater. Civ. Eng..

[22] Adnan AM, Lü C, Luo X, Wang J (2021). High-temperature rheological characteristics of asphalt binder incorporated with graphene oxide and predicting its rutting potential using response surface method. J. Mater. Civ. Eng..

[23] Tiwari B (2017). Mechanical properties of lightweight cellular concrete for geotechnical applications. J. Mater. Civ. Eng..

[24] Das D, Prakash P, Rout PK, Bhaladhare S (2021). Synthesis and characterization of superabsorbent cellulose-based hydrogel for agriculture application. Starch/Staerke.

[25] Suo F (2021). Preparation and characterization of biochar derived from co-pyrolysis of Enteromorpha prolifera and corn straw and its potential as a soil amendment. Sci. Total Environ..

[26] Liu Y (2020). Evaluating sulfur-free lignin as a sustainable additive for soil improvement against frost resistance. J. Clean. Prod..

[27] Salimi M, Motamedi E, Motesharezedeh B, Hosseini HM, Alikhani HA (2020). Starch-g-poly(acrylic acid-co-acrylamide) composites reinforced with natural char nanoparticles toward environmentally benign slow-release urea fertilizers. J. Environ. Chem. Eng..

[28] Alsohaimi I, Hafez IH, Berber MR (2021). Mechanically stable membranes of polyacrylic acid-grafted chitosan-functionalized carbon nanotubes with remarkable water storage capacity in sandy soils. J. Appl. Polym. Sci..

[29] Yao D, Qian G, Liu J, Yao J (2019). Application of polymer curing agent in ecological protection engineering of weak rock slopes. Appl. Sci..

[30] Ijaz, N., Dai, F., Meng, L., Rehman, Z. ur & Zhang, H. Integrating lignosulphonate and hydrated lime for the amelioration of expansive soil: A sustainable waste solution. *J. Clean. Prod.* **254**, 119985 (2020).

[31] Kim J-H, Oh D-K, Yoon Y-H (2013). Effects of super absorbent polymer on the growth of vine plants. J. Environ. Sci. Int..

[32] Huang, H. & Liu, L. Application of Water-soluble polymers in the soil quality improvement. in *Civil Engineering and Urban Planning 2012 - Proceedings of the 2012 International Conference on Civil Engineering and Urban Planning* (2012). doi:10.1061/9780784412435.022.

[33] Xi J, Zhang P (2021). Application of super absorbent polymer in the research of water-retaining and slow-release fertilizer. IOP Conf. Series Earth Environ. Sci..

[34] Wang X, Tao J (2019). Polymer-modified microbially induced carbonate precipitation for one-shot targeted and localized soil improvement. Acta Geotech..

[35] Azzam WR (2014). Utilization of polymer stabilization for improvement of clay microstructures. Appl. Clay Sci..

[36] Karim H, Al-Soudany K (2018). Improving geotechnical properties of clayey soil using polymer material. MATEC Web Conf..

[37] Sauceda M (2014). Soil-strength enhancements from polymer-infused roots. J. Geotech. Geoenviron. Eng..

[38] Ghimire S, Miramini S, Richardson M, Mendis P, Zhang L (2018). Role of dynamic loading on early stage of bone fracture healing. Ann. Biomed. Eng..

[39] Huang W, Liu Z, Zhou C, Yang X (2020). Enhancement of soil ecological self-repair using a polymer composite material. CATENA.

[40] Tao G (2021). Chemical stabilization of calcareous sand by polyurethane foam adhesive. Constr. Build. Mater..

[41] Zhou C, Zhao S, Huang W, Li D, Liu Z (2019). Study on the stabilization mechanisms of clayey slope surfaces treated by spraying with a new soil additive. Appl. Sci..

[42] Huang W (2021). New polymer composites improve silty clay soil microstructure: An evaluation using NMR. L. Degrad. Dev..

[43] Song, Z. *et al.* Laboratory and field experiments on the effect of vinyl acetate polymer-reinforced soil. *Appl. Sci.* **9**(1), 208 (2019)

[44] Yang CD, Lu SG (2021). Effects of five different biochars on aggregation, water retention and mechanical properties of paddy soil: A field experiment of three-season crops. Soil Tillage Res..

[45] Li L, Yuan Z, Li F (2019). Changes in soil aggregates composition stabilization and organic carbon during deterioration of alpine grassland. IOP Conf. Series Earth Environ. Sci..

[46] Ning, L., Shi, H., Zhou, H. & Liu, S. Quantitative characteristics of soil aggregates under different vegetations in upper reach of Minjiang River. *Chinese J. Appl. Ecol.* **16**(8), 1405–1410 (2005). 16262049

[47] Shi H, Shao HB (2012). Modifying transition matrix to evaluate soil quality A case study in Karst Region in the West-Southern China. Clean: Soil, Air, Water.

[48] Liu J, Shi B, Jiang H, Bae S, Huang H (2009). Improvement of water-stability of clay aggregates admixed with aqueous polymer soil stabilizers. CATENA.

[49] Nichols PD, Michael Henson J, Guckert JB, Nivens DE, White DC (1985). Fourier transform-infrared spectroscopic methods for microbial ecology: Analysis of bacteria, bacteri-polymer mixtures and biofilms. J. Microbiol. Methods.

[50] Gipson, K., Stevens, K., Brown, P. & Ballato, J. Infrared spectroscopic characterization of photoluminescent polymer nanocomposites. *J. Spectrosc.* **2015**, 1–9 (2015).

[51] Wiesner U, Reynolds N, Boeffel C, Spiess HW (1992). An infrared spectroscopic study of photo-induced reorientation in dye containing liquid-crystalline polymers. Liq. Cryst..

[52] Schjønning P, Lamandé M, Keller T, Labouriau R (2020). Subsoil shear strength – measurements and prediction models based on readily available soil properties. Soil Tillage Res..

[53] Wei S (2017). Impact of soil water erosion processes on catchment export of soil aggregates and associated SOC. Geoderma.

[54] Greathouse JA, Feller SE, McQuarrie DA (1994). The modified Gouy-Chapman theory: Comparisons between electrical double layer models of clay swelling. Langmuir.

[55] Liu J (2019). Topsoil reinforcement of sandy slope for preventing erosion using water-based polyurethane soil stabilizer. Eng. Geol..

[56] Razakamanantsoa AR, Djeran-Maigre I (2016). Long term chemo-hydro-mechanical behavior of compacted soil bentonite polymer complex submitted to synthetic leachate. Waste Manag..

